# The effect of customised and sham foot orthoses on plantar pressures

**DOI:** 10.1186/1757-1146-6-19

**Published:** 2013-05-17

**Authors:** Chris J McCormick, Daniel R Bonanno, Karl B Landorf

**Affiliations:** 1 Department of Podiatry, Faculty of Health Sciences, La Trobe University, Melbourne, Victoria 3086, Australia; 2 Lower Extremity and Gait Studies Program, Faculty of Health Sciences, La Trobe University, Melbourne, Victoria 3086, Australia

**Keywords:** Orthoses, Orthotic devices, Sham treatment, Kinetics

## Abstract

**Background:**

The effectiveness of foot orthoses has been evaluated in many clinical trials with sham foot orthoses used as the control intervention in at least 10 clinical trials. However, the mechanical effects and credibility of sham orthoses has been rarely quantified. This study aimed to: (i) compare the effects on plantar pressures of three sham foot orthoses to a customised foot orthosis, and (ii) establish the perceived credibility and the expected benefit of each orthotic condition.

**Methods:**

Thirty adults aged between 18 and 51 participated in this study. At 0 and 4 weeks, plantar pressure data were collected for the heel, midfoot and forefoot using the pedar^®^-X in-shoe system for the following five randomly assigned conditions: (i) shoe alone, (ii) customised foot orthosis, (iii) contoured polyethylene sham foot orthosis, (iv) contoured EVA sham foot orthosis, and (v) flat EVA sham foot orthosis. At the initial data collection session, each participant completed a Credibility/Expectancy Questionnaire (CEQ) to determine the credibility and expected benefit of each orthotic condition.

**Results:**

Compared to the shoe alone at week 0, the contoured polyethylene sham orthosis was the only condition to not significantly effect peak pressure at any region of the foot. In contrast, the contoured EVA sham orthosis, the flat EVA sham orthosis and the customised orthosis significantly reduced peak pressure at the heel. At the medial midfoot, all sham orthoses provided the same effect as the shoe alone, which corresponded to effects that were significantly different to the customised orthosis. There were no differences in peak pressure between conditions at the other mask regions, the lateral midfoot and forefoot. When the conditions were compared at week 4, the differences between the conditions were generally similar to the findings observed at week 0. With respect to credibility and expected benefit, all orthotic conditions were considered the same with the exception of the contoured polyethylene sham orthosis, which was perceived as being less credible and less likely to provide benefits.

**Conclusion:**

The findings of this study indicate that all of the sham orthoses tested provided the same effect on plantar pressures at the midfoot and forefoot as a shoe alone. However, the contoured EVA sham orthosis and the flat EVA sham orthosis significantly reduced peak pressure under the heel, which was similar to the customised orthosis. In contrast, the contoured polyethylene sham orthosis had no significant effect on plantar pressure and was comparable to the shoe alone at all regions of the foot. Hence, lower plantar pressures were found under the heel with some sham orthoses, but not with others. Importantly, participants perceived the polyethylene sham orthosis – the sham that had no effect on plantar pressure – to be the least credible orthosis and the least likely to provide benefits. This may be critical for the design of future clinical trials as it may introduce confounding effects that produce inaccurate results. These findings provide some evidence for the mechanical effects, treatment credibility and expected benefit of sham foot orthoses, which should be considered when they are used as a control intervention in a clinical trial.

## Background

Foot pain is commonly experienced in the general community – a recent systematic review of 31 studies calculated a pooled prevalence estimate in the adult population of 24% [1]. Foot orthoses are frequently used for the management of musculoskeletal disorders of the lower extremity [2-4] with the most common goal being to reduce symptoms and provide a beneficial functional outcome for the patient [4]. Although the specific mechanism of action of foot orthoses remains unclear, there is evidence that they provide significant effects on the mechanical function of the foot and lower limb [5-7].

Randomised controlled trials are often conducted to evaluate the effectiveness of foot orthoses for the management of various conditions. To achieve this, many trials have used a sham orthosis as a control or comparison intervention [8-17] (Table 1). Ideally, a sham intervention should not provide the same mechanical effects as a real intervention (i.e. the one being evaluated), and should provide as close to no effect as possible [18]. In addition, a sham intervention should be perceived as being equally credible when compared to the real intervention and participants should expect both interventions to provide similar benefits [18].

**Table 1:** Summary of studies that have used a sham orthosis as a control intervention when evaluating the effectiveness of foot orthoses

**Study**	**Participants**	**Foot orthosis**	**Sham orthosis**	**Outcomes**
Budiman-Mak et al., 1995 [8]	102 participants with rheumatoid arthritis.	Customised orthosis constructed from Rohadur with rearfoot and forefoot posting.	Molded thin leather shell with naugahyde top cover.	At 3 years, 25% of sham group had progression of their HAV angle compared with 10% for the treatment group (statistically significant).
Burns et al., 2006 [9]	154 participants with painful cavoid feet.	Customised orthosis constructed with a 3 mm polypropylene shell and full length Poron^®^ and Kashmeer top cover.	Full-length flat insole made from 3 mm latex foam with Kashmeer top cover.	At 3 months, foot pain and function scores (scale, 0–100) improved more with custom foot orthoses than with the sham, difference, 8.3 points and 9.5 points respectively (statistically significant). The customised orthosis reduced peak plantar pressures by 26% compared with 9% in the sham group (statistically significant).
Burns et al., 2009 [10]	61 participants with diabetes mellitus.	Customised orthosis constructed from a mesh of 8 mm Polylux, 8 mm Combilux, 2.3 mm Memorix, 3 mm Remember, and a 0.7 mm Calbino microfiber top cover (Thanner GmbH, Germany).	Removable flat, non-supportive 4 mm EVA shoe innersoles covered with a 0.7 mm Calbino top cover.	At 8 weeks, the customised and sham orthosis both provided similar improvements in foot pain and function scores. Compared to the sham group, customised group reduced peak pressure across the whole foot, 18% to 8% respectively (statistically significant).
Collins et al., 2009 [11]	179 participants with patellofemoral joint pain.	Prefabricated orthosis (Vasyli) made from low, medium or high density EVA. Some orthosis were heat moulded and had medial wedges and/or heel lifts added.	Full-length 3 mm flat EVA inserts, with no inbuilt arch or wedging.	At 6 weeks, the prefabricated foot orthosis produced significant improvements (19.8 mm) on the scale of global improvement compared to the sham orthosis (statistically significant). The foot orthosis provided moderate to marked improvement for 85% of participants compared to 58% for the sham orthosis.
Conrad et al., 1996 [12]	102 participants with rheumatoid arthritis.	Customised orthosis constructed from Rohadur with rearfoot and forefoot posting.	Molded thin leather shell with naugahyde top cover.	At 3 years, the customised and sham foot orthosis provided the same effects on disability and pain measures.
Finestone et al., 1999 [13]	404 participants from military infantry.	Two orthoses: (i) ‘soft’ customised polyurethane orthosis (grade 80 top layer, 60 middle layer, and 80 lower layer), and (ii) ‘semi-rigid’ customised polypropylene orthosis with rearfoot post.	Prefabricated full-length flat insole made of 3 mm polyolefin foam covered with Cambrelle^®^.	At 14 weeks, the ‘soft’ (10.7%) and ‘semirigid’ (15.7%) orthoses significantly reduced the incidence of the stress fractures compared to the sham orthosis (27%).
Landorf et al., 2006 [14]	135 participants with plantar fasciitis.	Two orthoses: (i) Customised semirigid polypropylene orthosis with heel post, and (ii) Formthotics^®^ prefabricated three-quarter length firm density orthosis made from polyethylene foam.	6 mm soft 120 kg/m^3^ EVA foam moulded to unmodified cast of participant’s foot. No top-cover. EVA shell was ground similarly to other orthoses, including being ground to approximately 1 mm thick under heel.	At 3 months, the customised and prefabricated orthoses produced significant improvements in function (scale, 0–100), 7.5 points & 8.4 points respectively, compared with the sham orthosis (statistically significant). Improvements in pain occurred in both orthotic groups compared with the sham, however these were not significant. At 12 months, no difference in pain and function was observed between orthotic groups.
Milgrom et al., 2005 [15]	404 participants from military infrantry.	Two orthoses: (i) ‘soft’ customised polyurethane orthosis (grade 80 top layer, 60 middle layer, and 80 lower layer), and (ii) ‘semirigid’ customised polypropylene orthosis with rearfoot post.	Prefabricated full-length flat insole made of 3 mm polyolefin foam covered with Cambrelle^®^.	At 14 weeks, no differences in subjective or objective measures of back pain were observed between the customised orthosis and sham groups.
Munteanu et al., 2009 [17]	140 participants with Achilles tendinopathy.	Customised orthosis constructed from polypropylene with a rearfoot post and covered with 2 mm Nora^®^ Lunasoft SL. Polypropylene thickness (3.0 mm, 4.0 mm or 4.5 mm) was determined by body mass and foot posture.	4.0 mm 90 km/m^3^ EVA with a 2 mm Nora^®^ Lunasoft SL top cover. Shell was minimally ground under heel.	Study in progress.
Novak et al., 2009 [16]	40 participants with rheumatoid arthritis.	Customised orthosis of three layers: (i) 6 mm cork (ii) 3 mm Plastazote^®^ and (iii) 2 mm Dynoshaum^®^.	‘Unshaped’ insole of three layers: (i) 6 mm cork (ii) 3 mm Plastazote^®^ and (iii) 2 mm Dynoshaum^®^.	No significant difference in pain, activity and plantar pressures was observed between the customised orthosis and sham groups.

At present, nine randomised trials [8-16] and one trial in progress [17] have used a sham orthosis as a control intervention when evaluating the effectiveness of foot orthoses. Importantly, there are substantial variations in the design parameters and materials used in the construction of the sham orthoses used in these trials. However, only two of the trials attempted to quantify the mechanical effects of the sham orthoses; both investigated their effects on plantar pressures [9,10]. With respect to the nine randomised trials that have been published, six of the trials found that sham orthoses provided similar clinical outcomes to real foot orthoses for pain [10-12,14-16]; although it should be noted that one trial conducted by Landorf et al. found that customised and prefabricated foot orthoses provided benefits in function over the sham when used for plantar fasciitis [14]. As the mechanical effects of the sham orthoses are often not quantified, it is not possible to rule out that the findings from some of these trials may have been due to mechanical effects from the sham orthoses.

Despite being used in clinical trials, evidence for the mechanical effects of sham foot orthoses is lacking. As such, a better understanding of the effects of sham foot orthoses will help guide their use in research and assist interpreting the findings of clinical trials that have used them to compare to an intervention being evaluated. The primary aim of this study was to evaluate the mechanical effects of customised and sham foot orthoses on plantar pressures. The secondary aim of this study was to evaluate the credibility and the expected benefit of each orthotic intervention.

## Methods

### Participants

Thirty adult participants, comprising 23 females (77%) and 7 males (23%) were recruited between May and September 2011 via poster advertising at a university. The study was approved by the Faculty Human Ethics Committee, Faculty of Health Science, La Trobe University (Ref FHEC11/10) and written informed consent was obtained from all participants. The sample size of 30 was selected as it was considered large enough to allow the use of parametric statistics during data analysis [19] and it is similar to previous plantar pressure studies we have recently conducted [20-22]. A power calculation was not performed due to the uncertainty surrounding the minimal important difference for plantar pressure changes.

All participants were aged 18 years or older and were able to walk household distances without the assistance of an aid. No specific foot posture or foot-related condition was required to participate in this study. Participants were excluded from the study if they had; a history of foot surgery, an inability to speak English, an existing lower limb injury, or a history of wearing foot orthoses within the past two years. In addition, to minimise expectation effects, 3rd and 4th year podiatry students (that had studied foot orthoses) were not allowed to participate. Participant characteristics and anthropometric measures, including the modified Foot Posture Index (FPI-6) [23] and normalised navicular height truncated [24] to determine the foot posture, were documented. Participant characteristics are displayed in Table 2.

**Table 2:** Participant characteristics (N = 30)

**Characteristic**	**Mean (SD)**	**Range**
Age in years	25.1 (9.63)	19 to 51
Height in m	1.70 (0.11)	1.53 to 1.92
Body mass in kgs)	68.2 (13.8)	44 to 96
Body mass index in kg/m^2^	23.4 (4.07)	17 to 36
Foot posture index	+4 (3.84)	−4 to +10
Normalised navicular height truncated	0.24 (0.04)	0.16 to 0.33
Time on feet in h/day	5.7 (2.0)	3 to 12

Note: The foot posture index uses six criterion-based observations, which are each scored on a 5-point scale (range −2 to +2); these are then summated to produce a final score which can range from −12 (very supinated) to +12 (very pronated) [23]. The normalised navicular height truncated is the ratio of navicular height relative to the truncated foot length – with a lower ratio indicative of a flatter-arched foot [24].

### Interventions

All participants wore standardised thin cotton socks with their most commonly used footwear during testing.

**Figure 1:**
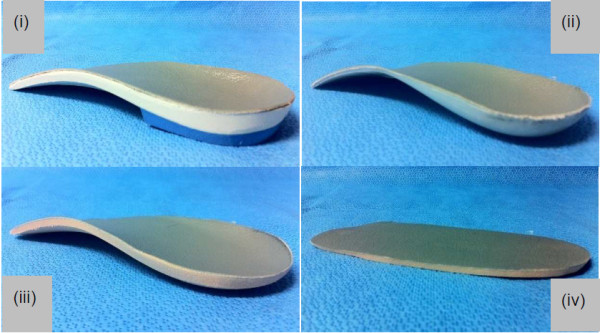
Posterior-medial view of the (i) customised foot orthosis, (ii) contoured polyethylene sham foot orthosis, (iii) contoured EVA sham foot orthosis, and (iv) flat EVA sham foot orthosis.

The five conditions analysed were: (Figure 1):

(i) Shoe alone (control),

(ii) Customised foot orthosis,

(iii)  Contoured polyethylene sham foot orthosis,

(iv) Contoured ethylene vinyl acetate (EVA) sham   foot orthosis,

(v)  Flat ethylene vinyl acetate (EVA) sham foot orthosis.

All feet were cast with the participant prone, using a commonly used technique to gain a neutral impression of the foot [25]. The customised foot orthosis was a modified Root style device balanced to the neutral calcaneal stance position as it is the most commonly prescribed orthosis by Australian and New Zealand podiatrists [25]. The orthotic shell thickness was either 3.0 mm, 4.0 mm or 4.5 mm polypropylene which was determined by each participant’s bodyweight and foot posture [17]. Participants with a neutral or pronated foot (FPI > 0) and bodyweight under 75 kg were prescribed a 4.0 mm polypropylene shell, while participants with a bodyweight 75 kg or over were prescribed a 4.5 mm shell. Participants with a cavoid foot (FPI < 0) were issued a 3.0 mm shell if their weight was less than 75 kg, while a 4.0 mm shell was issued if their weight was greater than 75 kg. Each customised foot orthosis was manufactured by a commercial orthotic laboratory (Footwork Podiatry Laboratory Pty Ltd, Melbourne, Australia) using a computer-aided design and computer-aided manufacturing process (CAD-CAM), whereby each orthosis was moulded against a positive cast that was milled from a timber composite block.

Following this, the two contoured sham orthoses were moulded against the same positive cast as the customised foot orthosis. One of the moulded sham devices was made from 1 mm polyethylene. The other moulded sham device was made from 3 mm EVA (90 kg/m^2^). These materials were chosen as both were expected to collapse under minimal force, thus providing minimal effect on plantar pressures (see Additional file 1: Figures S1 and S2 for basic information on the deformation characteristics of the foot orthoses). Both of these devices received minimal modification post-moulding, and the contoured orthosis had minimal grinding under the heel. The final flat sham orthosis was made from 3 mm EVA (90 kg/m^2^) and was only bevelled at the anterior margin just proximal to the metatarsophalangeal joint line. The contoured [14,17] and flat EVA sham foot orthosis [10] were also selected as similar devices have been used in previous randomised controlled trials or trials in progress. All sham and customised orthoses were covered with the same thin vinyl material.

### Procedures

Plantar pressure data were captured using the pedar^®^-X in-shoe system (Novel GmbH, Munich, Germany), which has been shown to be both an accurate and reliable measuring system for plantar pressures [26-28]. The pedar^®^-X system comprises 99 capacitance sensors arranged in a matrix that are embedded within a thin, flexible insole approximately 2 mm thick. Plantar pressure data were captured at a sampling rate of 50 Hz. The insoles were calibrated using the trublu^®^ calibration device (novel gmbh, Munich, Germany) prior to data collection.

Plantar pressure data were collected initially (i.e. Week 0) following the issue of the orthotic conditions. Participants were advised they were receiving four pairs of foot orthoses, with no additional information being provided. The five conditions (shoe alone and four orthotic conditions) were analysed in random order to minimise potential sequencing effects. Participants were blinded to the conditions being tested at the initial data collection session. Investigators were not blinded due to the difficulty in concealing each orthotic condition. In each test condition, the pedar^®^-X insole was placed on top of the orthosis and was zeroed prior to the first walking trial as per the manufacturer’s guidelines (novel gmbh, Munich, Germany). Following a two minute acclimatisation period of standing and walking, the participants completed four walking trails along an eight metre walkway for each condition, with each trial timed with a stopwatch to control for walking speed. If a trial did not fall within 5% of the original walking time it was repeated to minimise the effect of altered walking speed on plantar pressures [29]. The mean walking speed across all trials was 4.39 (±0.49) km/h. Only the middle four steps of each walking trial were included to minimise the effects of acceleration and deceleration. A total of 16 steps per participant per condition were included in the analysis.

At the initial data collection session, and after the 2-minute period of acclimatisation to each orthotic condition, participants completed a Credibility/Expectancy Questionnaire (CEQ) to determine each orthotic condition’s perceived credibility and expectancy to provide benefits [30]. At the time of completing the CEQ, the participants were yet to handle or see the orthoses. The CEQ consists of six questions that relate to either treatment credibility or expectancy. Participants rate four questions on a 9-point Likert scale and two questions are rated as a percentage score ranging from 0-100%. Higher Likert and percentage scores indicate that a treatment is perceived as being more credible and likely to provide a benefit. The CEQ has been shown to have good reliability and internal consistency [30].

Once the initial plantar pressure and CEQ data were collected, participants were then provided with the devices so they could begin wearing them for the ensuing four weeks. Participants were given instructions to wear each orthotic condition for an equal amount of time over the ensuing four week period. After four weeks, participants returned for a second data collection, where plantar pressure data were collected for each condition as per the protocol used at the initial data collection session. Similar to the initial data collection session, participants were blinded to the conditions being tested at the four week data collection session. The CEQ questionnaire was not administered at the four week session.

### Data analysis

The primary outcomes were peak pressure, contact area, and maximum force under the heel, midfoot and forefoot at zero and four weeks. The secondary outcome measures consisted of the contact time under the whole foot and the CEQ.

Plantar pressure data were analysed using the Novel-win program (version 20.3.30) and Novel percent masks were applied to each individual footprint. The heel mask consisted of the proximal 30% of the foot length, the midfoot mask consisted of the middle 30% of the foot length and the forefoot mask consisted of the distal 40% of the foot length [22]. The heel and midfoot masks were further bisected into medial and lateral halves. The forefoot mask consisted of the 1st metatarsophalangeal joint (1st MTPJ) region, lateral forefoot (including the 2nd through 5th metatarsophalangeal joint regions) and hallux. The lateral toes were excluded from analysis due to the previously reported high variability and low yield of plantar pressure data in this region [22].

Statistical analysis was performed using the Statistical Program for the Social Sciences (SPSS) Version 19 (SPSS inc, Chicago, Illinois) computer program. If data were not normally distributed it was transformed prior to analysis. One-way analysis of variance (ANOVA) with a Bonferroni-adjusted post hoc test was used for comparison of the means between test conditions. Statistical significance for hypothesis tests was set at the conventional level of *p* < 0.05.

## Results

### Week 0

There were a number of significant peak pressure, maximum force and contact area differences between the 5 conditions. Contact time did not vary between the conditions, or sessions at 0 and 4 weeks, so it can be assumed that participants walked at a consistent speed. Therefore, any differences observed can be attributed to individual conditions being analysed.

#### Medial heel

Compared to the control condition (i.e. the shoe alone), maximum force was significantly less (7-9%) with all orthotic conditions, while peak pressure was significantly less with the customised foot orthosis (13%), contoured EVA sham orthosis (13%) and flat EVA sham orthosis (11%) (Table 3). Comparison between the different orthotic conditions established that peak pressure was significantly less with the contoured EVA sham orthosis (9%) and the customised foot orthosis (8%) compared to the contoured polyethylene sham orthosis. There were no significant differences observed in contact area for any condition.

**Table 3:** Mean values (SD) and percentage change for the medial and lateral heel at week 0 (N = 30)

**Medial heel**									
	**Peak pressure (kPa)**			**Maximum force (%BW)**			**Contact area (cm**^**2**^**)**		
**Insert**	**Mean (SD)**	**% change**	***p*-value**	**Mean (SD)**	**% change**	***p*-value**	**Mean (SD)**	**% change**	***p*-value**
Shoe only (control)	215.5 (52.3)	n/a	n/a	38.2 (6.4)	n/a	n/a	19.7 (1.8)	n/a	n/a
Customised foot orthosis	187.8 (40.3)	−13%*^^^	<0.001	35.5 (6.4)	−7%*	0.004	20.0 (1.7)	+1%	0.495
Contoured polyethylene	204.5 (48.8)	−5%^#+^	0.180	35.5 (6.6)	−7%*	0.041	19.7 (1.8)	0%	1.000
Contoured EVA	186.7 (41.9)	−13%*^^^	<0.001	34.8 (5.7)	−9%*	<0.001	19.9 (1.8)	+1%	0.879
Flat EVA	190.8 (44.8)	−11%*	0.001	35.1 (6.2)	−8%*	0.008	19.9 (1.7)	+1%	0.230
**Lateral heel**									
	**Peak pressure (kPa)**			**Maximum force (%BW)**			**Contact area (cm**^**2**^**)**		
**Insert**	**Mean (SD)**	**% change**	***p*-value**	**Mean (SD)**	**% change**	***p*-value**	**Mean (SD)**	**% change**	***p*-value**
Shoe only (control)	213.9 (50.5)	n/a	n/a	37.8 (6.7)	n/a	n/a	19.4 (1.8)	n/a	n/a
Customised foot orthosis	198.8 (49.3)	−7%	0.251	40.0 (7.3)	+6%	0.134	19.7 (1.8)	+2%	0.319
Contoured polyethylene	209.3 (46.9)	−2%^+^	1.000	39.4 (6.9)	+4%	0.470	19.5 (1.8)	+1%	1.000
Contoured EVA	191.8 (39.5)	−10%*^^^	<0.001	38.6 (6.5)	+2%	1.000	19.7 (1.8)	+2%	0.319
Flat EVA	196.5 (52.3)	−8%*	0.015	40.1 (7.2)	+6%*	0.036	19.6 (1.8)	+1%	0.568

* Mean difference significant at the 0.05 level (Bonferroni adjusted) compared to the shoe only condition.

^#^ Mean difference significant at the 0.05 level (Bonferroni adjusted) compared to the customised orthosis.

Mean difference significant at the 0.05 level (Bonferroni adjusted) compared to the flat EVA sham orthosis.

^+^ Mean difference significant at the 0.05 level (Bonferroni adjusted) compared to the contoured EVA sham orthosis.

^^^ Mean difference significant at the 0.05 level (Bonferroni adjusted) compared to the contoured polyethylene orthosis.

#### Lateral heel

Compared to the control condition (i.e. the shoe alone), peak pressure was significantly less under the lateral heel with the contoured EVA sham orthosis (10%) and the flat EVA sham orthosis (8%). Significantly greater maximum force (6%) was also observed with the flat EVA sham orthosis (Table 3). When the orthotic conditions were compared, peak pressure was significantly less with the contoured EVA sham orthosis (8%) compared to the contoured polyethylene sham orthosis. No significant differences were observed in contact area between the conditions.

#### Medial midfoot

Compared with all other conditions, peak pressure was significantly greater under the medial midfoot with the customised foot orthosis (13-18%) (Table 4). No significant differences for peak pressure were observed between the sham orthoses.

**Table 4:** Mean values (SD) and percentage change for the medial and lateral midfoot at week 0 (N = 30)

**Medial midfoot**									
	**Peak pressure (kPa)**			**Maximum force (%BW)**			**Contact area (cm**^**2**^**)**		
**Insert**	**Mean (SD)**	**% change**	***p*-value**	**Mean (SD)**	**% change**	***p*-value**	**Mean (SD)**	**% change**	***p*-value**
Shoe only (control)	104.2 (32.1)	n/a	n/a	8.1 (8.0)#	n/a	n/a	14.2 (7.1)	n/a	n/a
Customised foot orthosis	119.7 (33.3)	+15%*^†+^	0.017	15.0 (7.6)#	+86%*^†+^^	<0.001	23.3 (3.5)	+65%*^†+^^	<0.001
Contoured polyethylene	98.2 (28.8)	−6%^#^	1.000	8.3 (8.0)#	+3%^#†+^	1.000	15.7 (7.6)	+11%^#†+^	0.054
Contoured EVA	101.6 (26.0)	−2%^#^	1.000	11.4 (8.1)#	+42%*^#^^	<0.001	19.3 (6.6)	+36%*^#^^	<0.001
Flat EVA	104.3 (26.6)	0%^#^	1.000	12.0 (7.9)#	+49%*^#^^	<0.001	20.0 (6.5)	+41%*^#^^	<0.001
**Lateral midfoot**									
	**Peak pressure (kPa)**			**Maximum force (%BW)**			**Contact area (cm**^**2**^**)**		
**Insert**	**Mean (SD)**	**% change**	***p*-value**	**Mean (SD)**	**% change**	***p*-value**	**Mean (SD)**	**% change**	***p*-value**
Shoe only (control)	121.7 (29.8)	n/a	n/a	20.8 (5.4)	n/a	n/a	23.5 (2.6)	n/a	n/a
Customised foot orthosis	126.0 (26.3)	+4%	1.000	25.4 (5.7)	+22%*^+^^	<0.001	24.0 (2.1)	+2%^^^	0.166
Contoured polyethylene	121.3 (23.5)	0%	1.000	22.4 (5.6)	+8%*^#†^	0.023	23.5 (2.4)	0%^#†+^	1.000
Contoured EVA	118.3 (23.7)	−3%	1.000	23.7 (4.5)	+14%*^#^	<0.001	24.0 (2.0)	+2%^^^	0.441
Flat EVA	115.0 (22.5)	−5%^#^	0.090	23.9 (4.5)	+15%*^^^	<0.001	24.0 (2.1)	+2%^^^	0.524

# Data transformed prior to determining significance.

* Mean difference significant at the 0.05 level (Bonferroni adjusted) compared to the shoe only condition.

^#^ Mean difference significant at the 0.05 level (Bonferroni adjusted) compared to the customised orthosis.

^†^ Mean difference significant at the 0.05 level (Bonferroni adjusted) compared to the flat EVA sham orthosis.

^+^ Mean difference significant at the 0.05 level (Bonferroni adjusted) compared to the contoured EVA sham orthosis.

^^^ Mean difference significant at the 0.05 level (Bonferroni adjusted) compared to the contoured polyethylene orthosis.

With the exception of the contoured polyethylene sham orthosis, maximum force (42-86%) and contact area (36-65%) was significantly greater with all orthotic conditions compared to the control condition (i.e. the shoe alone). Comparison between the orthotic conditions demonstrated that maximum force was significantly greater with the customised foot orthosis (25-81%) compared with all sham orthoses. Furthermore, maximum force was significantly greater with the flat EVA sham orthosis (44%) and the contoured EVA sham orthosis (37%) compared with the contoured polyethylene sham orthosis (Table 4).

#### Lateral midfoot

Compared to the control condition (i.e. the shoe alone), maximum force was significantly greater with all orthotic conditions (8-22%), while no differences in peak pressure and contact area were observed (Table 4). However, when the orthotic conditions were compared significant differences in maximum force, peak pressure and contact area were observed. Maximum force was significantly greater with the customised foot orthosis (7-13%) compared with the contoured EVA sham orthosis and the polyethylene sham orthosis, while peak pressure was also greater with the customised foot orthosis (10%) compared with the flat EVA sham orthosis. Compared to the flat EVA sham orthosis, maximum force was significantly less (6%) with the contoured polyethylene sham orthosis. Contact area was significantly less with the contoured polyethylene sham orthosis (2%) compared to all other orthotic conditions.

#### 1st MTPJ

Compared to the control condition (i.e. the shoe alone), a significantly greater contact area was observed with the contoured polyethylene sham orthosis (6%) under the 1st MTPJ (Table 5). There were no statistically significant differences in peak pressure, maximum force or contact area between any of the orthotic conditions.

**Table 5:** **Mean values (SD) and percentage change for the** 1st **MTPJ, lateral forefoot and hallux at week 0 (N = 30)**

**1st MTPJ**									
	**Peak pressure (kPa)**			**Maximum force (%BW)**			**Contact area (cm**^**2**^**)**		
**Insert**	**Mean (SD)**	**% change**	***p*-value**	**Mean (SD)**	**% change**	***p*-value**	**Mean (SD)**	**% change**	***p*-value**
Shoe only (control)	179.7 (56.3)	n/a	n/a	16.5 (6.9)	n/a	n/a	10.0 (1.3)	n/a	n/a
Customised foot orthosis	176.2 (51.6)	−2%	1.000	17.1 (5.4)	+4%	1.000	10.6 (1.0)	+6%	0.425
Contoured polyethylene	176.2 (46.6)	−2%	1.000	16.7 (5.4)	+1%	1.000	10.7 (1.0)	+6%*	0.020
Contoured EVA	169.1 (49.7)	−6%	0.074	16.0 (5.8)	−3%	1.000	10.6 (1.0)	+5%	0.096
Flat EVA	171.3 (49.8)	−5%	1.000	16.7 (5.7)	+1%	1.000	10.7 (1.0)	+6%	0.061
**Lateral forefoot**									
	**Peak pressure (kPa)**			**Maximum force (%BW)**			**Contact area (cm**^**2**^**)**		
**Insert**	**Mean (SD)**	**% change**	***p*-value**	**Mean (SD)**	**% change**	***p*-value**	**Mean (SD)**	**% change**	***p*-value**
Shoe only (control)	204.8 (66.1)	n/a	0.194	30.8 (9.2)	n/a	n/a	18.3 (1.5)	n/a	n/a
Customised foot orthosis	191.6 (54.1)	−6%	0.194	30.1 (5.9)	−2%^^^	1.000	18.5 (1.7)	+1%	0.252
Contoured polyethylene	205.2 (56.2)	0%^†+^	1.000	32.9 (7.2)	+7%*^#†+^	0.048	18.5 (1.6)	+1%	0.444
Contoured EVA	193.3 (56.9)	−6%^^^	0.665	30.9 (7.7)	0%^^^	1.000	18.5 (1.7)	+1%	0.252
Flat EVA	191.8 (58.2)	−6%^^^	0.177	30.8 (6.9)	0%^^^	1.000	18.5 (1.7)	+1%	0.533
**Hallux**									
	**Peak pressure (kPa)**			**Maximum force (%BW)**			**Contact area (cm**^**2**^**)**		
**Insert**	**Mean (SD)**	**% change**	***p*-value**	**Mean (SD)**	**% change**	***p*-value**	**Mean (SD)**	**% change**	***p*-value**
Shoe only (control)	248.2 (58.9)	n/a	n/a	29 .0(4.8)	n/a	n/a	14.3 (1.2)	n/a	n/a
Customised foot orthosis	258.6 (79.8)	+4%	1.000	29.2 (5.3)	+1%	1.000	14.1 (1.3)	−1%	1.000
Contoured polyethylene	256.3 (91.0)	+3%	1.000	28.2 (5.8)	−3%	1.000	14.2 (1.2)	0%	1.000
Contoured EVA	259.4 (96.5)	+5%	1.000	28.9 (5.5)	0%	1.000	14.2 (1.3)	0%	1.000
Flat EVA	260.0 (94.8)	+5%	1.000	29.3 (5.4)	+1%	1.000	14.3 (1.1)	0%	1.000

* Mean difference significant at the 0.05 level (Bonferroni adjusted) compared to the shoe only condition.

^#^ Mean difference significant at the 0.05 level (Bonferroni adjusted) compared to the customised orthosis

^†^ Mean difference significant at the 0.05 level (Bonferroni adjusted) compared to the flat EVA sham orthosis.

^+^ Mean difference significant at the 0.05 level (Bonferroni adjusted) compared to the contoured EVA sham orthosis.

^^^ Mean difference significant at the 0.05 level (Bonferroni adjusted) compared to the contoured polyethylene orthosis.

#### Lateral forefoot

Compared to the control condition (i.e. the shoe alone), maximum force was significantly greater (7%) with the contoured polyethylene sham orthosis under the lateral forefoot (Table 5). In addition, comparison between the different orthotic conditions demonstrated that peak pressure was significantly reduced with the contoured EVA sham orthosis (6%) and the flat EVA sham orthosis (7%) compared to the contoured polyethylene sham orthosis. No significant changes in contact area were observed between all conditions.

#### Hallux

No significant differences were observed between the shoe and orthotic conditions for peak pressure, maximum force and contact area (Table 5).

### Week 4

The majority of relationships between the conditions at all regions of the foot were similar at weeks 4 as they were at week 0. Compared to the control condition (i.e. shoe alone), the following plantar pressure changes were statistically significant at week 0 but no longer observed at week 4: maximum force being reduced at the medial heel with the contoured polyethylene sham and customised orthosis; maximum force being greater under the lateral heel with the flat EVA sham orthosis; contact area being greater under the 1st MTPJ with the contoured polyethylene sham orthosis and maximum force being greater with the latter under the lateral forefoot. Compared to the control condition, the following plantar pressure changes were only statistically significant at week 4: maximum force being greater under the lateral heel with the customised foot orthosis (7%); and contact area being greater with the contoured EVA sham orthosis (6%) and the flat EVA sham orthosis (6%) at the 1st MPTJ. Specific details of plantar pressure changes and relationships between conditions at week 4 are provided for the heel (Table 6), midfoot (Table 7) and forefoot (Table 8).

**Table 6:** Mean values (SD) and percentage change for the medial and lateral heel at week 4 (N = 30)

**Medial heel**									
	**Peak pressure (kPa)**			**Maximum force (%BW)**			**Contact area (cm**^**2**^**)**		
**Insert**	**Mean (SD)**	**% change**	***p*-value**	**Mean (SD)**	**% change**	***p*-value**	**Mean (SD)**	**% change**	***p*-value**
Shoe only (control)	220.8 (54.0)	n/a	n/a	38.6 (6.9)	n/a	n/a	19.7 (1.8)	n/a	n/a
Customised foot orthosis	190.8 (37.2)	−14%*^^^	<0.001	36.8 (6.4)	−5%	0.072	20.0 (1.7)	+2%	0.292
Contoured polyethylene	211.5 (46.8)	−4%^#†+^	0.237	36.9 (6.3)	−4%	0.132	19.7 (1.7)	0%	1.000
Contoured EVA	192.8 (45.2)	−13%*^^^	<0.001	35.6 (6.4)	−8%*	<0.001	19.9 (1.7)	+1%	0.229
Flat EVA	198.8 (42.9)	−10%*^^^	<0.001	36.7 (6.7)	−5%*	0.008	19.9 (1.7)	+1%	0.229
**Lateral heel**									
	**Peak pressure (kPa)**			**Maximum force (%BW)**			**Contact area (cm**^**2**^**)**		
**Insert**	**Mean (SD)**	**% change**	***p*-value**	**Mean (SD)**	**% change**	***p*-value**	**Mean (SD)**	**% change**	***p*-value**
Shoe only (control)	221.2 (53.9)	n/a	n/a	38.3 (7.1)	n/a	n/a	19.4 (1.9)	n/a	n/a
Customised foot orthosis	206.4 (44.6)	−7%	0.444	41.1 (7.1)	+7%*^+^	0.015	19.6 (1.8)	+1%	0.455
Contoured polyethylene	216.0 (45.6)	−2%^†+^	1.000	40.0 (6.8)	+4%^+^	0.166	19.6 (1.8)	+1%	0.455
Contoured EVA	197.6 (41.4)	−11%*^^^	<0.001	38.0 (5.9)	−1%^#^^	1.000	19.5 (1.8)	+1%	1.000
Flat EVA	201.8 (42.7)	−9%*^^^	<0.001	39.4 (7.1)	+3%	0.745	19.6 (1.8)	+1%	0.865

* Mean difference significant at the 0.05 level (Bonferroni adjusted) compared to the shoe only condition.

^#^ Mean difference significant at the 0.05 level (Bonferroni adjusted) compared to the customised orthosis.

^†^ Mean difference significant at the 0.05 level (Bonferroni adjusted) compared to the flat EVA sham orthosis.

^+^ Mean difference significant at the 0.05 level (Bonferroni adjusted) compared to the contoured EVA sham orthosis.

^^^ Mean difference significant at the 0.05 level (Bonferroni adjusted) compared to the contoured polyethylene orthosis.

**Table 7:** Mean values (SD) and percentage change for the medial and lateral midfoot at week 4 (N = 30)

**Medial midfoot**									
	**Peak pressure (kPa)**			**Maximum force (%BW)**			**Contact area (cm**^**2**^**)**		
**Insert**	**Mean (SD)**	**% change**	***p*-value**	**Mean (SD)**	**% change**	***p*-value**	**Mean (SD)**	**% change**	***p*-value**
Shoe only (control)	106.5 (30.9)	n/a	n/a	8.2 (7.9)#	n/a	n/a	14.4 (7.1)	n/a	n/a
Customised foot orthosis	119.3 (32.1)	+12%*^†+^^	0.040	14.9 (7.5)#	+81%*^†+^^	<0.001	23.3 (3.9)	+62%*^†+^^	<0.001
Contoured polyethylene	102.5 (30.7)	−4%^#^	1.000	9.0 (8.4)#	+10%^#†+^	0.354	16.1 (7.5)	+11%^#†+^	0.486
Contoured EVA	104.5 (29.0)	−2%^#^	1.000	12.3 (7.8)#	+49%*^#^^	<0.001	20.6 (6.2)	+43%*^#^^	<0.001
Flat EVA	106.5 (29.6)	0%^#^	1.000	12.2 (7.7)#	+48%*^#^^	<0.001	20.5 (6.1)	+42%*^#^^	<0.001
**Lateral midfoot**									
	**Peak pressure (kPa)**			**Maximum force (%BW)**			**Contact area (cm**^**2**^**)**		
**Insert**	**Mean (SD)**	**% change**	***p*-value**	**Mean (SD)**	**% change**	***p*-value**	**Mean (SD)**	**% change**	***p*-value**
Shoe only (control)	125.6 (33.9)	n/a	n/a	21.5 (5.4)	n/a	n/a	23.5 (2.4)	n/a	n/a
Customised foot orthosis	131.4 (30.3)	+5%^†+^	1.000	25.4 (5.4)	+18%*^†+^^	<0.001	24.1 (2.0)	+3%^^^	0.131
Contoured polyethylene	125.4 (29.5)	0%	1.000	23.4 (5.4)	+9%*^#^	0.001	23.6 (2.3)	+1%^#^	1.000
Contoured EVA	119.2 (26.0)	−5%^#^	0.930	23.7 (4.7)	+10%*^#^	0.002	24.1 (2.2)	+2%	0.305
Flat EVA	120.6 (30.8)	−4%^#^	0.909	24.1 (5.3)	+12%*^#^	<0.001	24.1 (2.2)	+3%	0.216

# Data transformed prior to determining significance.

* Mean difference significant at the 0.05 level (Bonferroni adjusted) compared to the shoe only condition.

^#^ Mean difference significant at the 0.05 level (Bonferroni adjusted) compared to the customised orthosis.

^†^ Mean difference significant at the 0.05 level (Bonferroni adjusted) compared to the flat EVA sham orthosis.

^+^ Mean difference significant at the 0.05 level (Bonferroni adjusted) compared to the contoured EVA sham orthosis.

^^^ Mean difference significant at the 0.05 level (Bonferroni adjusted) compared to the contoured polyethylene orthosis.

**Table 8:** Mean values (SD) and percentage change for the 1st MTPJ, lateral forefoot and hallux at week 4 (N = 30)

**1st MTPJ**									
	**Peak pressure (kPa)**			**Maximum force (%BW)**			**Contact area (cm**^**2**^**)**		
**Insert**	**Mean (SD)**	**% change**	***p*-value**	**Mean (SD)**	**% change**	***p*-value**	**Mean (SD)**	**% change**	***p*-value**
Shoe only (control)	180.1 (51.8)	n/a	n/a	16.3 (6.6)	n/a	n/a	10.1 (1.1)	n/a	n/a
Customised foot orthosis	180.6 (47.2)	0%	1.000	17.6 (5.1)	+8%	0.658	10.7 (1.0)	+6%	0.090
Contoured polyethylene	178.2 (43.9)	−1%	1.000	16.4 (5.4)	+1%	1.000	10.7 (1.1)	+6%	0.104
Contoured EVA	176.2 (46.5)	−2%	1.000	16.2 (6.1)	0%	1.000	10.6 (1.0)	+6%*	0.016
Flat EVA	177.7 (43.6)	−1%	1.000	16.5 (5.3)	+2%	1.000	10.7 (1.0)	+6%*	0.037
**Lateral forefoot**									
	**Peak pressure (kPa)**			**Maximum force (%BW)**			**Contact area (cm**^**2**^**)**		
**Insert**	**Mean (SD)**	**% change**	***p*-value**	**Mean (SD)**	**% change**	***p*-value**	**Mean (SD)**	**% change**	***p*-value**
Shoe only (control)	212.1 (67.1)	n/a	n/a	32.2 (10.2)	n/a	n/a	18.4 (1.6)	n/a	n/a
Customised foot orthosis	200.8 (57.1)	−5%	1.000	31.1 (7.5)	−4%^^^	1.000	18.5 (1.7)	+1%	0.230
Contoured polyethylene	213.3 (65.0)	+1%^+^	0.984	33.4 (9.0)	+3%^#+^	1.000	18.5 (1.7)	+1%	0.230
Contoured EVA	200.3 (59.6)	−6%	0.504	30.8 (8.3)	−4%^^^	0.582	18.5 (1.7)	+1%	0.230
Flat EVA	204.8 (63.6)	−3%^^^	1.000	32.2 (9.2)	0%	1.000	18.5 (1.7)	+1%	0.733
**Hallux**									
	**Peak pressure (kPa)**			**Maximum force (%BW)**			**Contact area (cm**^**2**^**)**		
**Insert**	**Mean (SD)**	**% change**	***p*-value**	**Mean (SD)**	**% change**	***p*-value**	**Mean (SD)**	**% change**	***p*-value**
Shoe only (control)	250.2 (68.6)	n/a	n/a	29.0 (6.1)	n/a	n/a	14.2 (1.3)	n/a	n/a
Customised foot orthosis	268.1 (102.1)	+7%	0.779	29.0 (6.4)	0%	1.000	14.2 (1.2)	0%	1.000
Contoured polyethylene	253.6 (92.4)	+1%	1.000	28.4 (6.7)	−2%	1.000	14.3 (1.2)	+1%	1.000
Contoured EVA	254.0 (80.2)	+2%	1.000	29.5 (6.1)	+2%	1.000	14.3 (1.3)	+1%	1.000
Flat EVA	263.0 (96.8)	+5%	1.000	29.2 (7.2)	+1%	1.000	14.2 (1.3)	+1%	1.000

* Mean difference significant at the 0.05 level (Bonferroni adjusted) compared to the shoe only condition.

^#^ Mean difference significant at the 0.05 level (Bonferroni adjusted) compared to the customised orthosis.

^†^ Mean difference significant at the 0.05 level (Bonferroni adjusted) compared to the flat EVA sham orthosis.

^+^ Mean difference significant at the 0.05 level (Bonferroni adjusted) compared to the contoured EVA sham orthosis.

^^^ Mean difference significant at the 0.05 level (Bonferroni adjusted) compared to the contoured polyethylene orthosis.

### Credibility/expectancy questionnaire (CEQ)

The customised foot orthosis demonstrated higher mean values in treatment credibility and expected benefit of treatment compared to all other conditions for each of the six questions of the CEQ (Table 9). Compared to all other conditions, the contoured polyethylene sham orthosis produced the lowest mean scores for credibility and treatment expectancy across all domains. Compared to the customised foot orthosis, there was no significant difference in treatment credibility for both the contoured EVA sham orthosis and the flat EVA sham orthosis. The contoured polyethylene sham orthosis was significantly less credible than the customised foot orthosis at five of the six questions.

**Table 9:** Mean values (SD) for the credibility/expectancy questionnaire (CEQ) (N = 30)

	**Insert**			
	**Customised foot orthosis**	**Contoured polyethylene**	**Contoured EVA**	**Flat EVA**
Question	Mean (SD)	Mean (SD)	Mean (SD)	Mean (SD)
1. At this point, how logical does the treatment offered seem?	6.53 (1.43)	5.10 (2.01)*	6.17 (1.62)	5.87 (2.01)
2. At this point, how successfully do you think this treatment will be in benefiting you?	6.47 (1.41)	4.73 (2.07)*	5.90 (1.63)	5.63 (1.81)
3. How confident would you be in recommending this treatment to a friend who experiences similar problems?	6.27 (1.60)	4.73 (2.30)*	5.90 (1.83)	5.73 (2.17)
4. By the end of the treatment period, how much benefit do you think will occur?	60% (21%)	42% (30%)*	54% (24%)	51% (27%)
5. At this point, how much do you really feel that the treatment will benefit you?	6.03 (1.56)	4.67 (2.22)	5.87 (1.74)	5.27 (2.00)
6. By the end of the treatment period, how much benefit do you really feel will occur?	58% (22%)	41% (31%)*	54% (25%)	49% (25%)

* Mean difference significant at the 0.05 level (Bonferroni adjusted) compared to the customised foot orthosis.

## Discussion

Sham foot orthoses are often used as a control intervention in clinical trials investigating the effectiveness of foot orthoses; however their mechanical effects and credibility have rarely been quantified. While a sham intervention is not considered a true placebo, it should provide as close to no effect as is attainable [31]. In this study, the contoured polyethylene sham orthosis was the only sham condition to have a minimal effect on plantar pressures in all regions of the foot when compared to the control condition (i.e. the shoe alone). Accordingly, not all of the sham foot orthoses tested in this study provided minimal mechanical effects.

However, although some of the sham foot orthoses provided similar effects to the customised foot orthosis in some regions of the foot, no sham condition provided the same effects as the customised foot orthosis across the entire foot. As such, although the sham orthoses evaluated in this study do provide some mechanical effects, they do not provide the same general effects as a customised orthosis (i.e. they do function as a sham intervention). Of importance, though, is that some of the sham orthoses behaved similarly to the customised orthosis in a few regions of the foot (i.e. plantar pressure mask regions) – this is of significance for future research.

The most pertinent example of this was in the medial heel region where the contoured EVA sham orthosis and the flat EVA sham orthosis provided significant reductions in peak pressure (10% and 13% respectively), which were similar to the customised orthosis (13%). This finding may be of interest in orthotic research for plantar heel pain, although it is uncertain whether this magnitude of the peak pressure reduction under the heel is clinically significant. Interestingly, silicon heel inserts – which we did not study – provide similar reductions [20,32], and in a randomised trial by Pfeffer et al. these inserts provided therapeutic benefits for people with plantar fasciitis over a relatively short-term period of 8 weeks [33]. With this in mind, the contoured EVA sham orthosis and the flat EVA sham orthosis we evaluated may have some impact on clinical trials that evaluate the effectiveness of foot orthoses for plantar heel pain/plantar fasciitis where such a device is used as a control intervention.

A more direct example of this is a randomised trial by Landorf et al., which compared a contoured EVA sham orthosis – similar to the one we tested – to a prefabricated orthosis and a customised orthosis for plantar fasciitis [14] (Table 1). They found that the prefabricated and customised foot orthoses provided short-term benefits (up to 3 months) compared with the sham orthosis (i.e. the real orthoses provided beneficial effects compared to the sham orthosis). However, when this finding is considered with the previous paragraph, it does not support the argument that the magnitude of plantar pressure reduction we observed under the heel is sufficiently large to be clinically significant. Clearly, these issues are complex and are not easily understood within the confines of our current knowledge.

It remains unknown what effects sham devices have on plantar pressures in the heel over a longer term (because of material breakdown), but we suggest caution when using the contoured EVA sham orthosis or the flat EVA sham orthosis in trials involving conditions where a reduction in peak pressures under the heel may be considered potentially beneficial. To illustrate this point further, the trial by Landorf et al. discussed above, found that sham and real foot orthoses provide similar long-term benefits (12 months) in pain and function for people with plantar fasciitis [14], although this most likely reflects the natural course of plantar fasciitis, rather than any substantial orthotic material changes [14].

As the mechanical effects of the sham orthosis used in the Landorf et al. trial weren’t quantified, it is uncertain to what extent they may have negated any benefits provided by the real foot orthoses. Despite this, it cannot be assumed that the mechanical effects provided by the sham orthoses in this study can be generalised to the sham device used in the Landorf et al. trial – as the latter was thicker (6 mm vs. 3 mm), had no top-cover and the EVA shell under the heel was ground to a minimal thickness (approximately 1 mm thick). Nevertheless, to minimise the uncertainty surrounding the effect of a sham orthosis, future trials that use a sham orthosis should either quantify the sham’s mechanical effects or choose a sham that is known to provide as minimal effect as possible under the region of the foot that is under evaluation – our data from this study will assist in this process.

The similar effects provided by the contoured EVA sham orthosis and the flat EVA sham orthoses in reducing peak pressure at the heel could be attributed to a combination of the shock attenuating capabilities of EVA and its ability to conform to the foot and redistribute forces. Our findings differ slightly from Goske and colleagues whereby insole conformity was the more important factor in reducing peak pressure rather than material properties [34]. Although conformity of an insole is likely to be important, our findings suggest that material selection contributes to plantar pressure changes as the polyethylene sham orthosis, which had the same topography as the contoured EVA sham orthosis, provided different effects under the heel.

As discussed previously, the sham foot orthoses generally had the same mechanical effects as a shoe alone in the midfoot and forefoot, while also being different to a customised foot orthosis in the medial midfoot. The midfoot findings can be considered particularly interesting as one of the major mechanisms for how foot orthoses achieve their effects is through changes in plantar pressures that are applied to the medial midfoot [22]. These findings are consistent with those of previous studies that indicate that foot orthoses increase plantar pressures in the midfoot [20-22]. As the sham devices had no significant effect on midfoot and forefoot pressures, all could be considered as viable sham orthoses in clinical trials where a minimal effect on midfoot and forefoot pressures are desirable.

Interestingly, the contoured EVA sham orthosis and the flat EVA sham orthosis were also perceived as being an equally credible intervention as the customised foot orthosis and participants expected that they were likely to provide similar benefits. In contrast, the contoured polyethylene sham orthosis, the only sham device to provide similar mechanical effects to the control condition (i.e. the shoe alone), was perceived as a less credible treatment and less likely to provide the same benefits as the other orthoses evaluated. This perceived reduction in credibility and expectancy is likely to be due to the polyethylene shell being relatively thin and, under the loading conditions of this study, the compressibility of the polyethylene was likely to be negligible. The contoured polyethylene sham orthosis was one-third the thickness of both EVA devices and it is possible that this reduced material thickness was more likely to bottom out under the force of bodyweight. Furthermore, because of its hard, non-compressible nature, it does not have the same shock attenuating properties as EVA. The lack of credibility finding is important for researchers when considering the style of sham orthosis they will use in their clinical trial as treatment credibility and expectancy is considered important in the early stages of treatment [30,35]. From a practical standpoint in a clinical trial, reduced credibility and expectancy of an intervention may introduce confounding effects, such as resentful demoralisation [36].

The findings of this study need to be viewed in light of some limitations. First, there are some technical issues regarding in-shoe pressure measurement that must be considered [37-39]. Although the pedar^®^-X has been shown to be a valid and reliable plantar pressure system it can only record forces applied perpendicular to the pressure sensors [26,27,40]. Accordingly, the shear component of forces acting at the orthosis-foot interface is unable to be determined [37,38]. Furthermore, the contact surface of an orthosis is curvilinear but the sensors are calibrated when placed flat. As a result of these issues, it is possible that inherent measurement error occurs, but the magnitude of any such error is currently unknown. In addition, potential accuracy errors have been shown to exist with plantar measuring systems when measuring contact area, otherwise referred to as spatial resolution [39,41,42]. Despite such limitations, in-shoe pressure measuring systems are considered the best available method for measuring forces acting between the orthosis and the foot [37,38] and it is commonly used when evaluating the mechanical effects of foot orthoses [20-22]. Second, despite the CEQ being a valid and reliable method of determining a person’s perception of a treatment’s credibility and expected effectiveness it was not intended to be used to compare different treatments within a single participant [30]. In this study, as the CEQ was completed after a short period of acclimatisation to each orthotic condition within the same data collection session, there is the likelihood that a participant’s perception regarding the credibility and expected effectiveness of each orthosis was influenced by their experiences with other orthotic conditions worn within the same session. In addition, as the participant’s completed the CEQ without viewing or handling the orthoses, it remains unclear how the appearance of the devices may have influenced their perceptions of credibility and expectations of benefit. Only a randomised controlled trial could remove these confounding effects. Third, caution is required when generalising this study’s findings to clinical trials that have used sham orthoses as a comparator intervention as it is likely that any variations in the sham’s design parameters and materials will provide different mechanical effects on the foot, as demonstrated in this study. Therefore, it is recommended that the similarities and differences of the sham orthoses used in this study, compared to those used in clinical trials, be considered. Accordingly, blanket categorisation of all sham orthoses is not encouraged until further evaluation is conducted. Fourth, while the sham orthoses did provide some effect on plantar pressures, we are unable to predict the consequences of this to other biomechanical parameters, such as kinematics, although it has been proposed that kinematic and kinetic effects associated with foot orthoses occur via changes in mechanical loading of the foot [22,43]. Finally, and most importantly, it remains unclear how these plantar pressure changes may influence clinical outcomes.

## Conclusion

The results of this study indicate that different sham orthoses provide different mechanical effects on the foot. The contoured polyethylene sham orthosis was the only sham device to provide similar effects as the shoe alone condition (i.e. the control) at all regions of the foot. In contrast, the contoured EVA sham orthosis and the flat EVA sham orthosis significantly reduced peak pressures at the heel, which was similar to the customised orthosis. For the midfoot and forefoot, all of the sham orthoses evaluated did not significantly alter plantar pressures. The contoured polyethylene sham orthosis was found to be the most appropriate device when a small mechanical effect across all regions of the foot is desired; however this device was the least credible of all the sham orthoses, which may lead to confounding effects in clinical trials. Therefore, when selecting a sham orthosis for a clinical trial it is necessary to establish the features of the sham, as not all sham orthoses provide the same mechanical effects or are perceived as being equally credible.

## Competing interests

The authors declare that they have no competing interests.

## Authors’ contributions

All authors were involved in the preparation of the study procedures. CJM collected the plantar pressure data and CJM and DRB were involved in data analysis. All authors were responsible for the preparation of the manuscript. The material within has not been and will not be submitted for publication elsewhere. All authors read and approved the final manuscript.

## Supplementary Material

Additional file 1: Figure S1 Example of deformation testing of a foot orthosis (in the figure below the contoured polyethylene sham foot orthosis is being tested). **Figure S2.** Force required for maximum deformation of the midpoint of the medial aspect of the foot orthosis. Click here for file
