# Supplementary material for: The effect of customised and sham foot orthoses on plantar pressures
Source: J Foot Ankle Res. 2013 May 17;6:19. doi: 10.1186/1757-1146-6-19 (PMC3663766; doi:10.1186/1757-1146-6-19)
Supplement: Additional file 1: Figure S1 — Example of deformation testing of a foot orthosis (in the figure below the contoured polyethylene sham foot orthosis is being tested). Figure S2. Force required for maximum deformation of the midpoint of the medial aspect of the foot orthosis. [file 1757-1146-6-19-S1.docx]

**Supplementary file 1**

**Figure 1:** Example of deformation testing of a foot orthosis (in the figure below the contoured polyethylene sham foot orthosis is being tested)


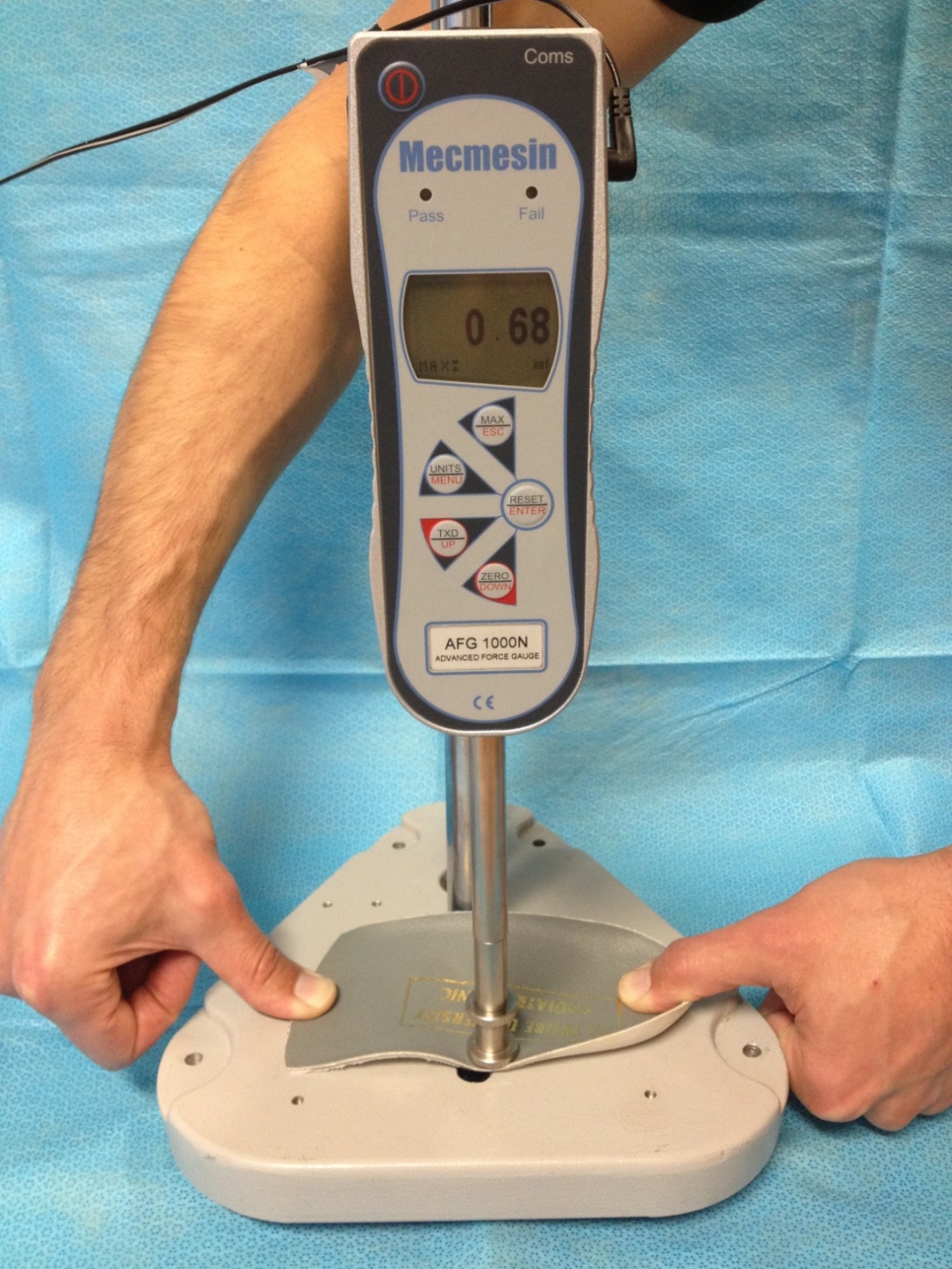


Notes: The force gauge (Mecmesin Advanced Force Gauge, Mecmesin Ltd, West Sussex, England) was applied to the midpoint of the medial aspect of the foot orthosis until it touched the base plate of the loading device (maximum deformation). The orthosis was stabilised proximally and distally, as indicated, by the investigator. The device was zeroed before all tests.

**Figure 2:** Force required for maximum deformation of the midpoint of the medial aspect of the foot orthosis

Force in Kg

Notes: The customised foot orthosis used in deformation testing was made from 3.0 mm polypropylene. Results were an average of three tests. The investigators provide the following information as a guide only, and although a consistent protocol was used no validity or reliability testing was conducted.
